# Efficacy and Safety of Infliximab Versus Adalimumab in Adult Subjects With Moderate to Severe Ulcerative Colitis: A Systematic Review and Meta-Analysis

**DOI:** 10.7759/cureus.61547

**Published:** 2024-06-02

**Authors:** Ahmed Salman, Mohamed A Salman, Ahmed Elewa, Asmaa M Awwad

**Affiliations:** 1 Department of Internal Medicine, Faculty of Medicine, Cairo University, Cairo, EGY; 2 Department of Surgery, KasrAlAiny School of Medicine, Cairo, EGY; 3 Department of General, Laparoscopic, and Hepato-Pancreato-Biliary (HPB) Surgery, National Hepatology and Tropical Medicine Research Institute, Cairo, EGY; 4 Department of Radiation Oncology, National Cancer Institute, Cairo University, Cairo, EGY

**Keywords:** meta-analysis, real-world experience, infliximab, adalimumab, ulcerative colitis

## Abstract

Ulcerative colitis (UC) is an inflammatory disorder affecting the colon, and typically, during the disease course, the condition may exacerbate, relapse, and remit. One of the most successful lines for inducing and maintaining clinical remission in subjects with UC is biological therapy with anti-tumor necrosis factor α (anti-TNF) agents, including adalimumab (ADA) and infliximab (IFX). This meta-analysis is an attempt to obtain complementary information driven by real-world experience (RWE) concerning the efficacy and safety of two of the most popular anti-TNFs in treating UC. This is a systematic review and meta-analysis of RWE studies comparing ADA and IFX as naïve anti-TNF agents for the treatment of subjects with UC. Studies were obtained by searching Scopus, Google Scholar, the Cochrane Central Register of Controlled Trials, Embase, and the PubMed Central databases. Patients treated with IFX showed significantly higher induction responses. No statistically significant difference was found in the comparison of response in the maintenance treatment period. Higher overall adverse events were related to IFX treatment, with serious adverse events that were nonsignificantly higher in the ADA-treated group. In conclusion, IFX demonstrated significantly higher induction responses compared to ADA in patients with moderate-to-severe UC. IFX was associated with higher overall adverse events, whereas serious adverse events were non-significantly higher in the ADA-treated group. IFX may be favored as a first-line agent for its induction efficacy, and the choice between IFX and ADA should be individualized based on comprehensive clinical evaluation.

## Introduction and background

Ulcerative colitis (UC) is an inflammatory disorder affecting the colon, and it is typically featured by inflammation of the bowel mucosa that usually begins in the rectum and proceeds steadily to the proximal portion of the colon. During the disease course of UC, typically, the condition exacerbates, relapses, and remits [1]. As a result, the fundamental objective of treatment is to establish and maintain clinical remission. One of the most successful lines for inducing and maintaining clinical remission in patients with UC is biologic therapy with anti-tumor necrosis factor α (anti-TNF) agents, including adalimumab (ADA) and infliximab (IFX), alone or in combination with immunomodulators. These agents are particularly important in patients who are refractory to traditional medical treatment. It has also been shown to reduce the risk of hospitalization and surgery [2].

IFX is the first anti-TNF agent approved for cases with moderate to severe Crohn’s disease (CD). It is a chimeric monoclonal antibody. ADA was developed later, and it is a humanized monoclonal antibody. Both drugs have different routes and rates of administration. This variability is likely associated with the variable efficacy and safety of the two agents [3]. However, there is still an absence of head-to-head treatment comparison trials that would help to recommend one drug over the other. Therefore, several indirect meta-analysis studies were conducted to compare both drugs based on the randomized controlled trials (RCTs) that compare each agent against a placebo [4-11].

Currently, several studies that present and document real-world experience (RWE) regarding the efficacy and safety of various anti-TNFs exist. RWE refers to clinical data collected outside of traditional RCTs and other controlled research environments. These data are gathered from various sources, such as electronic health records (EHRs), claims and billing activities, product and disease registries, and patient-generated data. RWE provides insights into how treatments perform in broader, more diverse populations and under real-world conditions, reflecting routine clinical practice rather than the controlled conditions of RCTs. This type of evidence is crucial for understanding long-term outcomes, safety profiles, and treatment adherence, making it invaluable for complementing the findings from RCTs and enhancing clinical decision-making. RWE has been assimilated into the recently published British Society of Gastroenterology guidelines and the consensus of the United Arab Emirates on the diagnosis and treatment of IBD. These guidelines ensure comparable anti-TNF-related UC clinical remission rates in both RWE and RCT sources [12,13].

This study, to the best of our knowledge, is the first direct meta-analysis incorporating data from RWE studies comparing ADA against IFX in variable outcomes. This meta-analysis is an attempt to obtain complementary information driven by RWE concerning the efficacy and safety of two of the most popular anti-TNFs in treating UC.

## Review

This is a systematic review and meta-analysis that was conducted per the Preferred Reporting Items for Systematic Reviews and Meta-Analyses (PRISMA) guidelines that were followed when performing this research [14].

Selection strategy and criteria

The search for relevant studies was conducted by two independent researchers (A.S. and M.A.S.). The articles addressing the study question were searched using the recognized database electronic resources. These were the Cochrane Central Register of Controlled Trials (CENTRAL), Embase, Scopus, Google Scholar, and the PubMed Central database.

The keywords “anti-TNF,” “infliximab,” “adalimumab,” “biologic-naïve,” “first line,” and “ulcerative colitis,” were used during the electronic search. The search in the described databases was limited to original articles available as full text in the English language. We further checked the references of the obtained article to preclude any missing work.

Inclusion and exclusion criteria

Original articles presenting data concerning the efficacy and/or safety of at least infliximab and adalimumab for the management of biologic-naïve patients with UC were candidate for the research.

Reviews, commentaries, and letters to the editor were excluded. The retrieved articles were screened. Studies that did not compare infliximab and adalimumab in terms of clinical outcome, efficacy, and safety; studies that did not discriminate cases of biologic-naïve ulcerative colitis from anti-TNF-exposed cases; studies that did not delineate data of patients with ulcerative colitis from those with CD; and studies involving pediatric subjects were also not included.

Data extraction, collection, and analysis

The included articles were dedicatedly reviewed. The data concerning the efficacy and/or safety of at least infliximab and adalimumab for the management of biologic-naïve patients with UC were extracted and recorded. The included research works were assessed for the bias encountered using the Cochrane Collaboration tool for assessing the risk of bias.

Summary measures

The study objectives were the difference between the two drugs in the induction and maintenance response, mucosal remission, and the change in the partial Mayo score (pMS). The secondary outcomes were the difference in safety-related items, including overall adverse events (AEs) and serious adverse events (SAEs). 

Statistical analysis

The retrieved data were recorded and tabulated. The statistical analysis was performed using version 28 of the SPSS statistical software (IBM Corp., Armonk, NY, USA). The categorical data were expressed as odds ratios and 95% confidence intervals (CIs), and the numerical variables were compared using the means difference (MD) and the standard error (SE). An odds ratio of more than 1 indicates a higher occurrence of the tested outcome in the ADA group, and a ratio of less than 1 indicates the reverse. The Review Manager Software (RevMan version 5.4, the Cochrane Collaboration, London, United Kingdom) was used to perform the pooled analysis of IFX versus ADA hazard ratios. A hazard ratio of more than 1 indicates higher risk in the IFX group and vice versa. The risks of bias in the included studies were also assessed using the RevMan software. The relative weight of each study in the relevant analysis was presented. The heterogeneity of each meta-analysis was assessed using the I2 statistic's indication of data heterogeneity and the Q statistics. According to the data homogeneity, the random or fixed-effect models were used for the analysis.

Results

**Figure 1:**
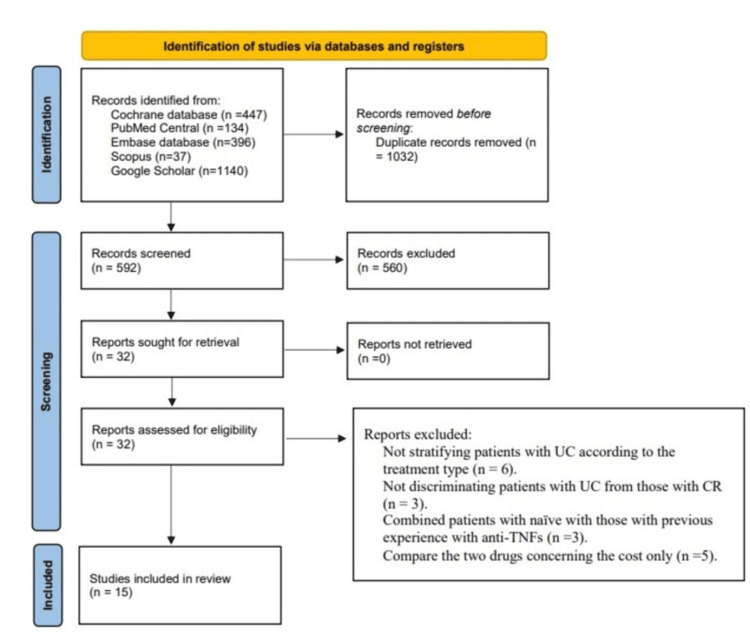
PRISMA 2020 study selection flow chart for new systematic reviews that included searchers of databases and registers

Initially, researching the electronic resources gave 1,624 records. The remaining records after adjusting for the duplications were 592. After checking the articles’ titles and abstracts, an additional 560 articles were not included. Reading the full texts of the remaining 32 articles resulted in the exclusion of 17 articles. Finally, 15 articles were eligible for this study [15-29]. They were all observational studies, of which only three were prospective studies [15,20,24]. The review flow chart is shown in Figure 1.

The included studies were published between 2010 and 2023. Subjects with inflammatory bowel diseases (UC and CD) or UC only who were prescribed IFX and ADA and were biologic-naïve were the study population. The included studies' sample sizes ranged from 25 [20] to 3340 [22], with a total population of 8,070. Of them, 3,035 (37.6%) patients were treated with ADA, and 5,035 (62.4%) were treated with IFX. Data concerning the baseline patients’ demographic and studies’ characteristics are presented in Table 1.

**Table 1:** Baseline patients’ demographic data and study characteristics RS: retrospective study, PS: prospective study, pMS: partial Mayo score

Study	N		Males			Age			Design	Country	Assessment of response	Outcome
	ADA	IFX	ADA		IFX	ADA	IFX					
Gies et al. (2010) [15]	25	28	14		18	34±12	31±10		PS	Canada	pMS	Response rates and long-term outcomes
Ma et al. (2015) [16]	36	66	20		42	37.6±12.8	40.1±13		RS	Canada	pMS + endoscopy	Secondary loss of response
Sandborn et al. (2016) [17]	380	424	218		241	39±12.7	39.3±12.9		RS	USA	pMS	Comparative effectiveness
Singh et al. (2016) [18]	288	1112	150		150	42±14	43±16		RS	USA	pMS or endoscopy	Comparative effectiveness and safety
Singh et al. (2017) [19]	104	171	48		79	38.7±12.8	37.8 ±12.1		RS	Denmark	pMS or endoscopy	Comparative effectiveness and safety
Mizoshita et al. (2017) [20]	15	10	12		5	46.9 (20-84)	52.9 (31-69)		PS	Japan	pMS + endoscopy	Preference and reasons for drug choice
Pouillon et al. (2019) [21]	34	126	15		65	34 (26.2-43.2)	34.3 (25.4-49.8)		RS	France	pMS	Treatment persistence rates
Chen et al. (2019) [22]	1473	1867	733		1315	42.67±15.69	39.24±17.81		RS	USA	NA	Treatment persistence rates
Kitayama et al. (2020) [23]	41	30	32			46 (30-61)			RS	Japan	pMS	Short- and long-term efficacy
Han et al. (2020) [24]	232	630	146	399		39.1±16.3		37.6±16.3	RS	Korea	pMS + endoscopy	Comparative effectiveness
Mohammed et al. (2020) [25]	30	70	NA			34.2±12.1			PS	Iraq	pMS + Endoscopy	Comparative effectiveness
Lee et al. (2021) [26]	30	83	22		49	39±14.1	38.2±18.2		RS	Korea	pMS + endoscopy	Efficacy and long-term outcomes
Cassinott et al. (2022) [27]	56	73	NA			NA			RS	Italy	pMS + endoscopy	Comparative effectiveness
Bulu et al. (2022) [28]	13	13	NA			42.04±16.8			RS	Turky	Truelove-Wittz activity index + endoscopy	response rates
Dalal et al. (2023) [29]	278	332	130		147	36 (26-50)	34 (26-51)		RS	USA	pMS + endoscopy	Long-term drug survival related to non-response and adverse effects

The baseline characteristics were generally comparable in the two arms. However, the IFX-treated patients had a significantly more severe disease in the studies of Ma et al. [16], Lee et al. [26], Dalal et al. [29], and Cassinotti et al. [27], longer follow-up period in the study of Singh et al. [18], higher use of steroids and other immunomodulators in the study of Chen et al. [22], shorter disease duration in the studies of Han et al. [24] and Cassinotti et al. [27], longer previous use of steroids and other immunomodulators in the work of Han et al. [24], lower use of steroids and other immunomodulators in the study of Dalal et al. [29], and different ethnic groups (lower in White and higher in Black/African) in the study of Sandborn et al. [17].

**Figure 2:**
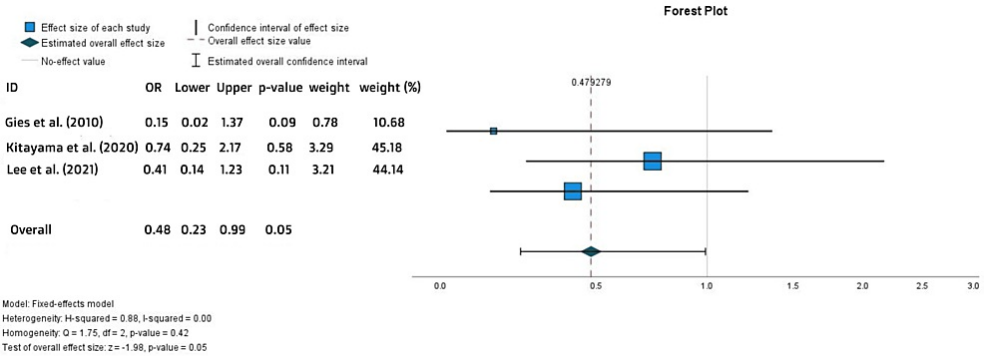
Forest plot for the induction response References: [15,23,26]

The outcome of each study is shown in Table 1. The primary outcomes varied among the studies but generally focused on the efficacy and/or safety of the drugs, including response rates, long-term outcomes, comparative effectiveness, treatment persistence rates, and specific adverse events. The induction response was described in three studies [15,23,26]. Their meta-analysis demonstrated a considerably lower rate of an induction response in the ADA-treated patients (OR = 0.48, 95% CI = 0.22-0.99, p = 0.05). The value of I2 was 0, which ensures the reliability of the results (Figure 2).

**Figure 3:**
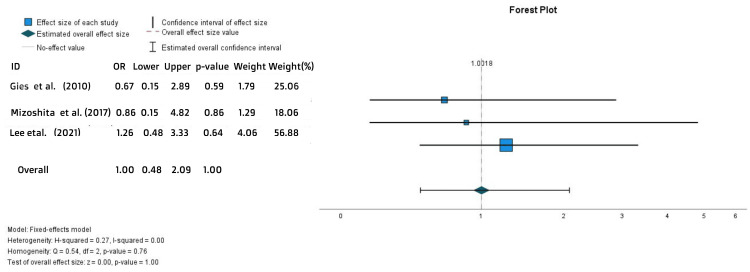
Forest plot for the maintenance clinical response References: [15,20,26]

Concerning the drugs’ efficacy during the maintenance phase, there was an equal clinical response (OR = 1, 95% CI = 0.48-2.09, p = 1) (Figure 3) and a higher rate of clinical remission in ADA-treated patients.

**Figure 4:**
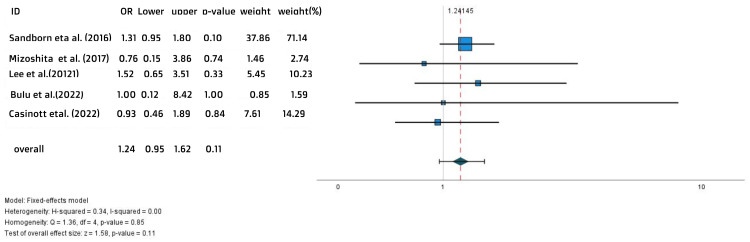
Forest plot for the maintenance clinical remission References: [17,20,26-28]

This difference did not reach the level of significance (OR = 1.24, 95% CI = 0.95-1.62, p = 0.11) (Figure 4).

**Figure 5:**
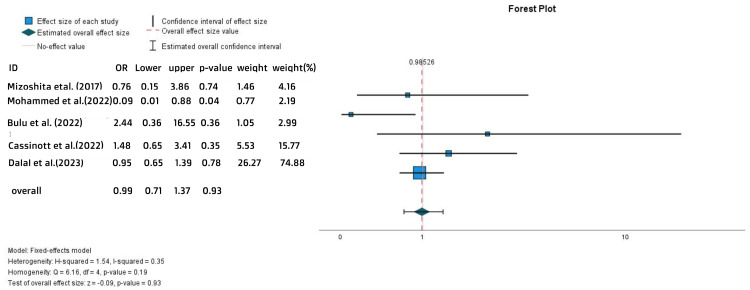
Forest plot for the mucosal healing References: [20,24,26-28]

The endoscopic findings as manifested in mucosal healing were quite equal in the two groups (OR = 0.99, 95% CI = 0.71-1.37, p = 0.93) (Figure 5).

**Figure 6:**
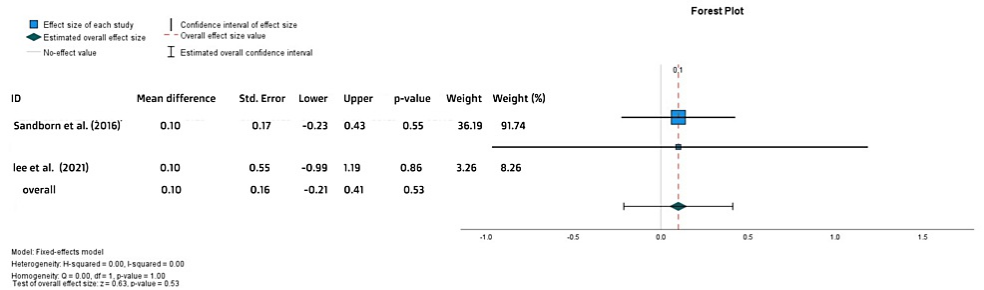
Forest plot for the pMS change References: [17,25]

The pMS change after treatment was reported in two studies only. Their work showed no statistically significant difference between the two arms (MD = 0.1, SE = 0.16, p = 0.53) (Figure 6).

**Figure 7:**
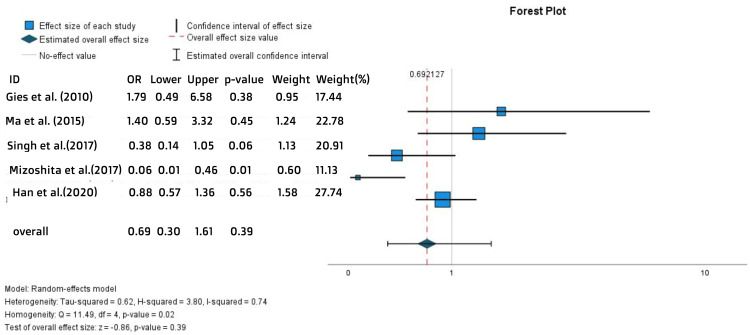
Forest plot for the new concomitant steroid use References: [15,16,19,20,24]

**Figure 8:**
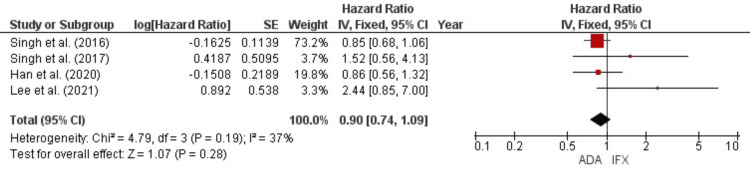
Forest plot for the hazards ratios of new steroid use References: [18,19,24,25]

No statistically significant difference was found in the new concomitant steroid use. This was evident from the pooled analysis of the percentages of new users (OR = 0.69, 95% CI = 0.3-1.61, p = 0.39) (Figure 7) and the hazards ratios of new use (HR = 0.9, 95% CI = 0.74-1.09, p = 0.28) (Figure 8). Evaluating the need for new concomitant steroid use provides insights into the efficacy and management of disease flares, which is crucial for both the induction and maintenance of remission.

**Figure 9:**
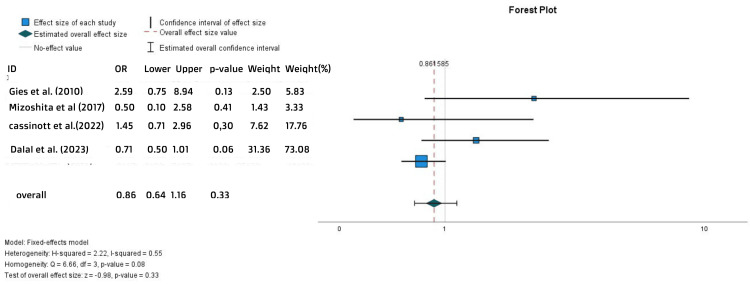
Forest plot for the steroid-free remission References: [15,20,26,28]

Moreover, no statistically significant difference was shown in the percentages of steroid-free remission in both groups (OR = 0.86, 95% CI = 0.64-1.16, p = 0.33) (Figure 9).

**Figure 10:**
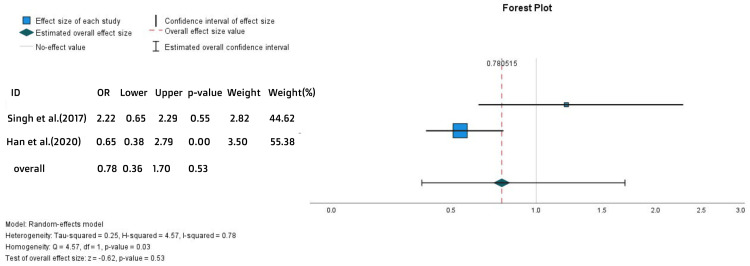
Forest plot for the UC-related hospitalization References: [19,24]

**Figure 11:**
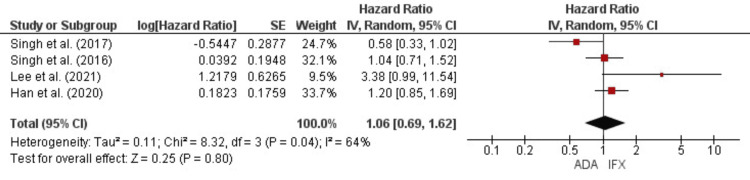
Forest plot for the hazards ratios of hospitalization References: [18,19,24,25]

There was no statistically significant difference in UC-related hospitalizations. This evidence was driven by the meta-analysis of the percentages of UC-related hospitalization (OR = 0.78, 95% CI = 0.35-1.7, p = 0.53) (Figure 10) and the hazards ratios of hospitalization (HR = 1.09, 95% CI = 0.69-1.62, p = 0.8) (Figure 11).

**Figure 12:**
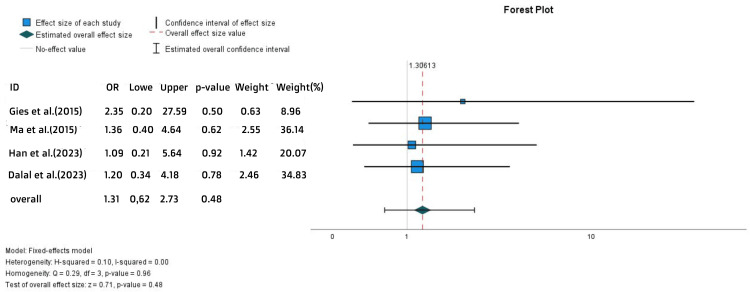
Forest plot for the colectomy References: [15,16,24,27]

No statistically significant difference was also found in the occurrence of colectomy, which serves as a crucial indicator of long-term disease management and treatment efficacy (OR = 1.31, 95% CI = 0.62-2.73, p = 0.48) (Figure 12).

**Figure 13:**
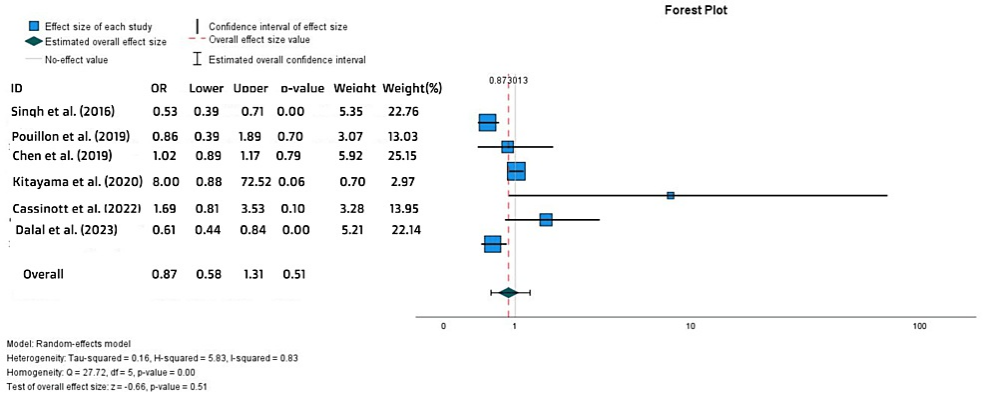
Forest plot for the treatment persistence References: [18,21-23,25,27]

**Figure 14:**
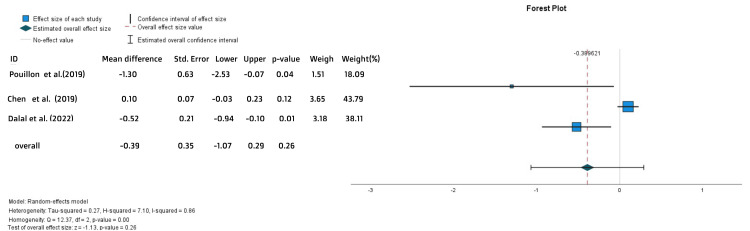
Forest plot for the time to treatment withdrawal References: [21,22,27]

No statistically significant difference was found in the rate of treatment persistence (OR = 0.87, 95% CI = 0.58-1.31, p = 0.51) (Figure 13) or the time to withdrawal (MD = -0.39, SE = 0.35, p = 0.26) (Figure 14).

Treatment persistence refers to the duration of time from the initiation to the discontinuation of therapy. It measures how long a patient continues to take a prescribed medication without interruption. In clinical research and practice, treatment persistence is an important metric as it provides insights into the real-world effectiveness, tolerability, and patient adherence to a given therapy.

**Figure 15:**
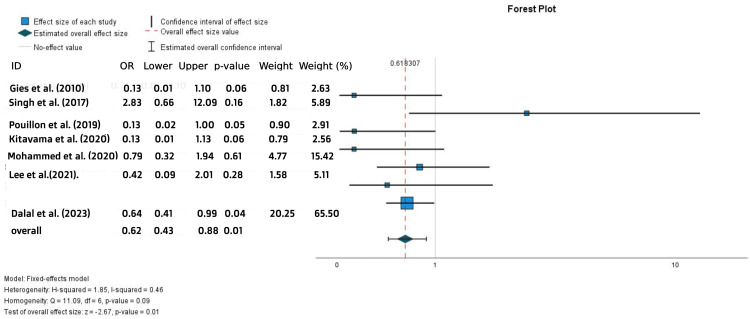
Forest plot for the AEs References: [1,2,19,21,23,25,28]

**Figure 16:**
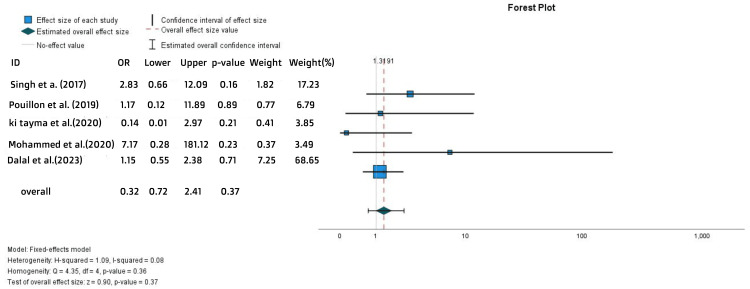
Forest plot for the serious AEs References: [2,19,21,23,28]

Significantly higher overall AEs were observed with IFX treatment compared to ADA (OR = 0.62, 95% CI = 0.43-0.88, p = 0.01) (Figure 15). However, most of these adverse events were not serious. By contrast, when comparing serious AEs (SAEs) only in the two groups, a trend toward a higher rate of SAEs was noted in the ADA-treated group, but this difference was not statistically significant (OR = 1.32, 95% CI = 0.72-2.41, p = 0.37) (Figure 16).

Risks of Bias

**Figure 17:**
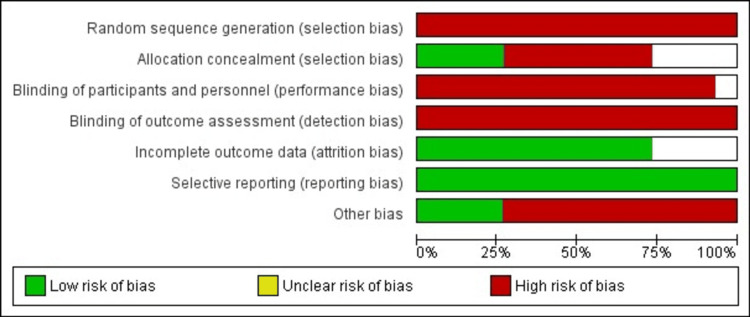
Risk of bias summary

The authors’ critical appraisal of the included studies’ methodological qualities was done and is displayed in Figure 17.

The primary bias in all the included research was embedded in the selection and performance bias, being non-RCTs with no blinding or randomization. The majority of the included articles were judged to be low-risk in terms of bias of incomplete outcome data (11/15; 73.3%) and selective reporting (15/15; 100%). Other biases were encountered in 11 studies (86.7%). These were baseline imbalance between groups, small sample size, using clinical judgment only for assessment of response and remission (partial Mayo score; pMS), and/or imbalance in the concomitant treatment.

Discussion

A significant portion of patients with ulcerative colitis are resistant to conventional medical care because it is a relapsing disease that is challenging to treat [3]. Anti-TNF therapy has been hailed as a paradigm-shifting method for treating patients with UC patients since its launch in the middle of the 2000s [29].

IFX and ADA constitute the mainstay of anti-TNF agents. IFX was introduced and approved first, followed by ADA. In this context, physicians who encounter patients with inflammatory bowel disease (IBD) during clinical practice need to identify the best choice for their patients, in terms of efficacy and safety.

In spite of the proven efficacy of both drugs in managing the disease activity of moderate-to-severe UC, there has been no head-to-head RCT comparing the two drugs yet. Due to the previously mentioned lack of RCTs, all existing systematic reviews and meta-analyses are indirect and do not evaluate comparison studies. The indirect methodology used to calculate the results against the placebo rather than each individual drug was criticized. The most reliable technique to compare the clinical efficacy and safety of biological therapy is through a direct comparative study of the two medications.

One cannot ignore the evidence that is driven by the recent RWE comparing ADA to IFX that could enhance clinical practice and help clinicians navigate a scenario in which the appropriate therapeutic options are adopted. Despite the fact that RCTs are the ideal research design to assess the efficacy and safety of two drugs, with a strong internal validity that is drawn from the highly selective and controlled design, there is still concern about the external validity (generalizability) of the obtained results. This point specifically impacts diseases that are known for ethnic diversity [30]. This is the case in UC, which is different in various populations with probable genetic causes. On the other hand, RWE, despite being ranked one step lower in evidence-based medicine, provides a design that promotes the generalizability of the obtained data compared to RCTs [31]. 

In this meta-analysis, we incorporated RWE studies that compare ADA to IFX and were not previously included in any pooled analysis.

This study's findings agree with the previous indirect meta-analyses that reported the superiority of IFX in the induction phase in terms of significantly better clinical response [4-11]. This superiority was particularly evident given that out of the three studies that assessed induction phase clinical response [15,23,25], two studies reported significantly higher baseline disease severity in the IFX group [23,25]. Although the remaining study showed significantly higher baseline use of immunosuppression in the IFX group [15], which could have played a role in the better response, this study reported that a multivariate regression test of the sociodemographic and combining medication treatment variables did not reveal any specific factors that could affect the induction response. Moreover, this study weighted only less than 11% of the analysis. This may be related to the difference in pharmacokinetics and bioavailability and route of administration.

In the present study, the IFX superiority has no longer been evident at the maintenance phase. No other significant differences were shown between both arms in this study regarding either of the efficacy parameters (maintained clinical response, maintained clinical remission, mucosal healing, pMS score change, and persistence on treatment). Notably, there was an equal clinical response (OR = 1) and quite equal mucosal healing in the two groups (OR = 0.99). These findings are supported by previously reported comparable efficacy of both drugs at the maintenance phase [4-11].

Concerning the safety outcome, there was no statistically significant difference between the two arms in steroid use and steroid-free remission. Interestingly, ADA was unexpectedly related to significantly fewer AEs than IFX. No cases, however, proceeded to death in either group. Moreover, no significant difference was demonstrated between the two arms in the occurrence of SAEs, hospitalization rate, or colectomy. The higher overall adverse events in the ADA group were not found in the previous meta-analysis comparing ADA to IFX, this may be attributed to their indirect comparative design. The recent direct meta-analysis that was conducted by Yang et al. [32] and pooled results from cohort studies comparing ADA to IFX in patients with CD also found significantly higher overall AEs in the IFX group and attributed this to the higher incidence of infusion or allergic reactions.

Overall, our meta-analysis demonstrates that IFX ranked first in the induction phase, but this superiority was lost during the maintenance phase. It was associated with higher overall AEs despite ADA treatment showing a higher trend toward serious AEs. Thus, the selection of either one is still a matter of debate. Particular concerns should be put into consideration and may tilt the odds toward one of the two drugs. First, some factors were described to affect the drug efficacy and/or safety. These were described by Laredo et al. [3] who presumed that the patient’s age, BMI, comorbidities, and extra-intestinal manifestations should be a concern during the choice of either drug. Finally, the patient’s preferences should not be ignored since the different administrations may affect the decision. IFX is given intravenously every eight weeks, whereas ADA is administered subcutaneously every two weeks only. Some may prefer ADA being less invasive, and others may prefer IFX being given at more distant intervals. The patient’s socioeconomic condition and the health insurance state in the healthcare system could be another determinant. IFX showed a lower cost per sustained clinical response than ADA in the treatment of moderately to severely active UC [33]. In the study of Petryszyn et al. [34], the authors found that although incremental cost-effectiveness ratios for all biologic agents were exceeding the threshold acceptability in Poland, the most cost-effective solution for naïve patients was IFX. Similar findings were reported by Yokomizo et al. [35] in the USA. On the contrary, Trigo-Vicente et al. [36] found that ADA was less costly than IFX in Italy. This discrepancy could be explained by that the acquirement cost of each medication relies on the decision-makers of every country. It is also influenced by the differences in healthcare systems and the policies of reimbursement among countries.

The present work is limited by the analysis of observational studies only. However, this could offer the results external validity. Moreover, some outcomes were assessed by the meta-analysis of two to three studies. Indeed, it has been reported that a number of two studies is sufficient for a statistically recognized meta-analysis in the condition that both studies were homogenous, and at least five studies are required for the same purpose if there was heterogeneity among studies. This was the case in almost all of our performed analyses. Only one analysis included two heterogeneous studies (pMS change). However, this was a complementary test since the clinical improvement was judged through the analysis of response and remission. This is the first meta-analysis that includes studies investigating the potential differences between two of the most commonly used first-line anti-TNFs for treating UC refractory to traditional medical treatment.

## Conclusions

The findings of this meta-analysis shed light on the comparative efficacy and safety profiles of IFX and ADA in the management of moderate-to-severe UC. While IFX demonstrated superiority in the induction phase, this advantage was not sustained during the maintenance phase, where both drugs exhibited comparable efficacy outcomes. Interestingly, while ADA was associated with fewer overall adverse events compared to IFX, comparing serious events alone showed that there was a trend towards a higher incidence of serious adverse events with ADA, although this difference was not statistically significant. The choice between IFX and ADA remains a matter of clinical judgment, taking into account patient-specific factors, such as disease severity, comorbidities, and patient preferences regarding treatment administration. Furthermore, considerations regarding cost-effectiveness and healthcare system dynamics should inform treatment decisions in individualized patient care pathways. Overall, this meta-analysis underscores the importance of tailored therapeutic approaches in optimizing outcomes for patients with UC refractory to conventional medical therapy.
